# Weak evidence of density dependent population regulation when using the ability of two simple density dependent models to predict population size

**DOI:** 10.1038/s41598-024-55533-4

**Published:** 2024-02-29

**Authors:** Demissew T. Gebreyohannes, Jeff E. Houlahan

**Affiliations:** Department of Biological Sciences, University of New Brunswick, Saint John, E2L 4L5 Canada

**Keywords:** Biological techniques, Ecology

## Abstract

The relative importance of density dependence regulation in natural population fluctuations has long been debated. The concept of density dependence implies that current abundance is determined by historical abundance. We have developed four models—two density dependent and two density independent—to predict population size one year beyond the training set and used predictive performance on more than 16,000 populations from 14 datasets to compare the understanding captured by those models. For 4 of 14 datasets the density dependent models make better predictions (i.e., density dependent regulated) than either of the density independent models. However, neither of the density dependent models is statistically significantly superior to density independent models for any of the 14 datasets. We conclude that the evidence for widespread density dependent population regulation in the forms represented by these two simple density-dependent models is weak. However, the density dependent models used here—the Logistic and Gompertz models—are simple representations of how population density might regulate natural populations and only examine density-dependent effects on population size. A comprehensive assessment of the relative importance of density-dependent population regulation will require testing the predictive ability of a wider range of density-dependent models including models examining effects on population characteristics other than population size.

## Introduction

One of the central issues in ecology has been assessing the relative importance of density dependent factors in regulating natural populations^1–7^. In the mid-1900’s Nicholson^8^ asserts that most populations are strongly density dependent regulated, implying that current population sizes are regulated by past population sizes due to the negative impact of population size on population growth rate^8,9^. It is widely accepted that population regulation cannot take place in the absence of density dependence^10–14^. Thus, regulation requires density-dependent negative feedback, in which small populations tend to increase while large populations decrease because of density^7^.

Peter Turchin has described 3 fundamental laws of population dynamics—first, that populations will grow or decline exponentially given a constant environment, second, that populations are self-limited, i.e. that there is a negative relationship between population growth rate and population density at high densities, and third, that there will be consumer-resource oscillations^15,16^. One reasonable implication of these laws is that natural populations will exhibit density-dependent regulation once they move beyond the initial colonization process. However, there is also evidence that environmental factors such as disturbance, temperature, and precipitation can have density independent effects on populations. The question of the relative importance of density-dependent versus density-independent factors remains unresolved^17,18^.

Traditional model selection in ecology has used model fit exclusively. Model selection is the process of comparing various competing models to identify the one that best fits the observed data. It is used to determine the most suitable model from a set of competing hypotheses by assessing how well each model fits the observed data^19,20^. Models are evaluated based on quantitative measures and information criteria such as the Akaike Information Criterion (AIC), Bayesian Information Criterion (BIC), p-values, and Mallows' Cp statistic^20^. These metrics allow us to rank and weigh the competing models, providing insights into their goodness of fit and complexity. Furthermore, model averaging can be employed to combine information from multiple models, leading to more robust parameter estimation and improved prediction accuracy. By incorporating various models, model averaging accounts for uncertainties and enhances the overall performance, particularly in cases where the true underlying model is uncertain or difficult to identify^21,22^.

Although density dependent may be expected in natural populations that persist for a long time, the methodology of detecting density dependence in real datasets has been the subject of controversy. Ecologists have spent a great deal of time and energy identifying suitable statistical methods for the detection of density dependence in long-term time series data^1,12,23–27^. Most involve choosing between density-dependent and density-independent models using traditional model selection techniques. There is no general consensus among ecologists about how to detect density dependent population regulation^24,25^.

The detection of density dependence has proven to be a thorny methodological problem. Traditionally negative relationships between population size and per capita population growth rate have been a putative diagnostic of density dependence^24,26^ but there is compelling evidence that such relationships can be found in populations that are not regulated by density^28^. The debate about how best to detect density dependence has been ongoing for several decades but has shed more heat than light, in part, because it is the wrong question. The more useful question is—how does the incorporation of density dependence into ecological models improve our understanding of population fluctuations? Our approach follows on the philosophy that understanding can only be demonstrated by prediction on independent data^29,30^.

Thus, we propose to use the predictive ability of models built using long-term population time series data to detect density dependence. We examined the predictive ability of simple population dynamic models to assess what we understand about the relationship between population size and per capita growth rate. Our objective is to use the predictive ability of density dependent models relative to density independent models to estimate how common and important density dependent population regulation is in natural populations.

## Results

### Density dependent versus density independent models

#### Mean prediction error

**Figure 1:**
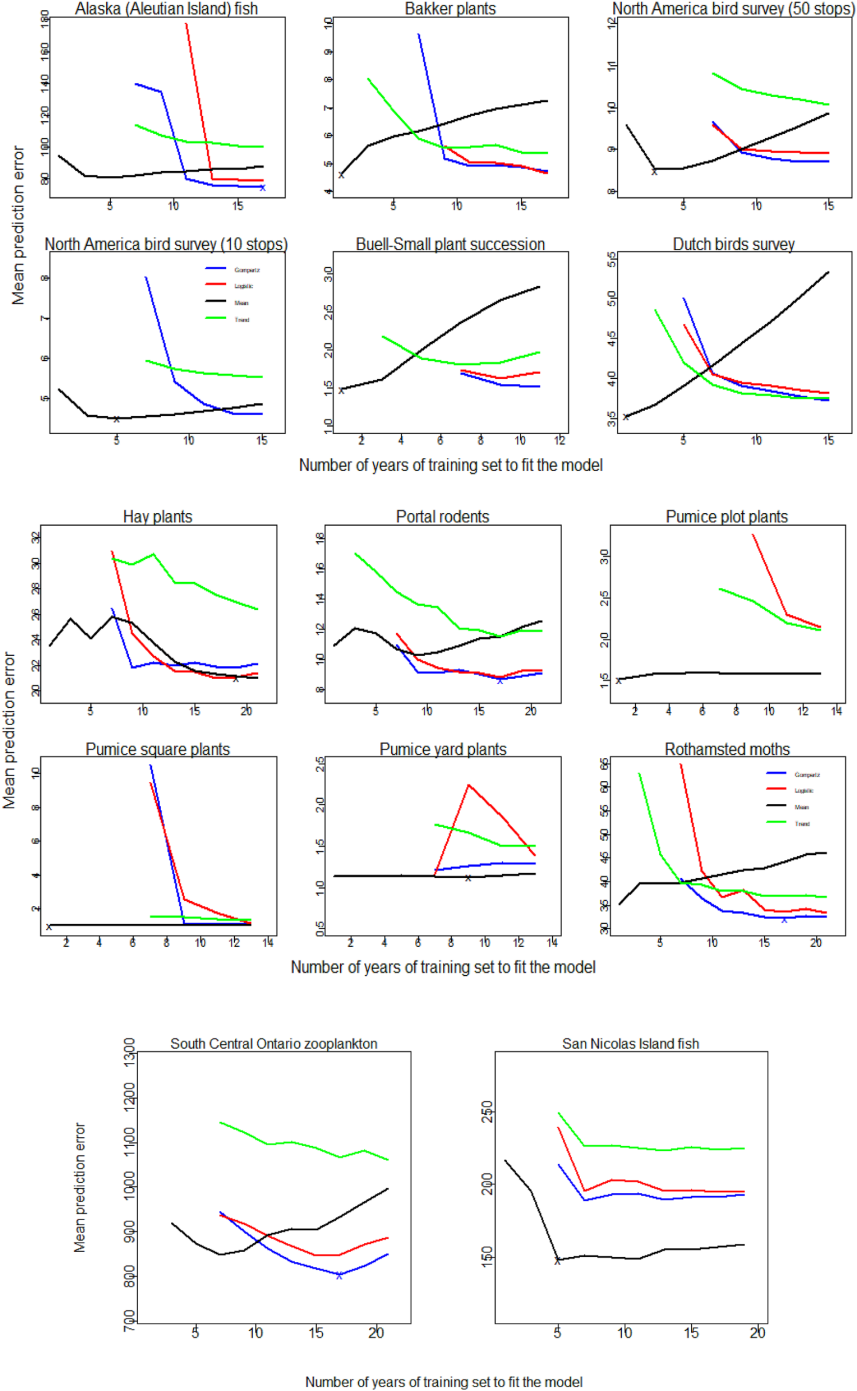
Mean absolute prediction error against the number of years of training set for each of 14 datasets. ‘x’ represents the minimum mean prediction error obtained by the best performing model. Note- Predictions can be made using one year of training data for the mean model but require at least three years of data for the other models. Where lines do not extend back to three years it is because the predictions were so poor they extended far beyond the scale of y-axis. Additionally, in cases where these models entirely resulted in a large prediction error across the training set, they were completely removed from the graph as in the case of North America bird survey (10 stops) datasets where the Logistic model was totally removed.

**Figure 2:**
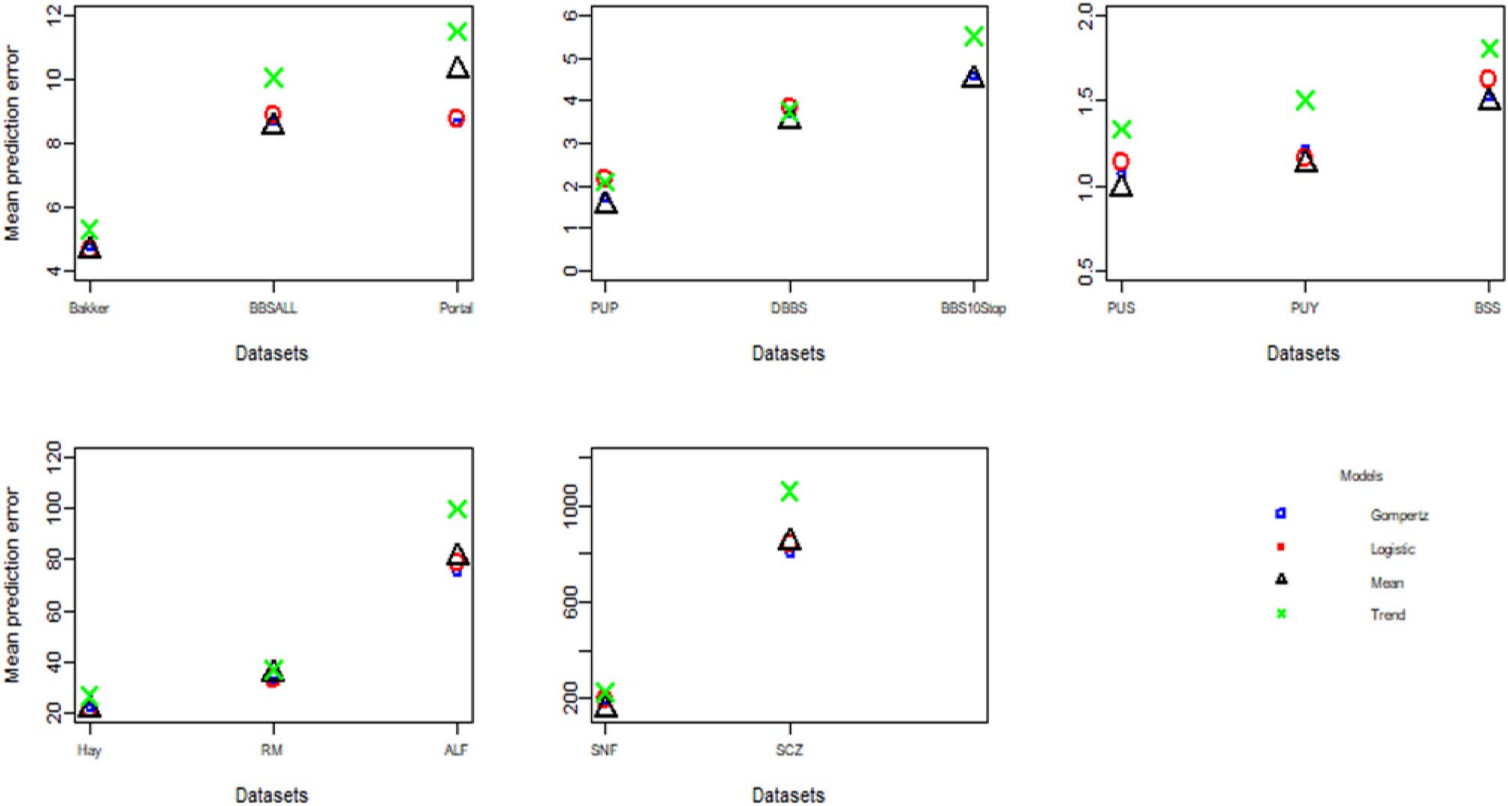
Mean absolute prediction error using ‘best’ training set size for each of the 14 datasets. Because of the range values along the y-axis differed among the 14 datasets, we grouped datasets with similar y-axis ranges.

We found the 'Mean' model had the smallest mean prediction error in 10 of 14 datasets, while the Gompertz model provided best mean prediction error for 4 of the 14 datasets (Figs. 1, 2). Thus, for ten of the fourteen datasets a density-independent model made the best predictions, on average. Often the Mean model that made the best predictions used small training sets (i.e. 1–5 years) implying that many populations fluctuated in a way that resembled a random walk or fluctuations around a recent short-term mean more than fluctuations around a long-term mean.

### Binomial and Chi-square test for density dependent and independent models

For thirteen of fourteen datasets, density independent models made superior predictions more often than density dependent models. In 7 out of 14 datasets, a binomial test showed that density-independent models had superior predictive ability statistically significantly more frequently than density-dependent models (Table 1). For six of the seven remaining datasets density-independent models had better predictive ability more often than density-dependent models but the difference wasn’t statistically significant.

**Table 1:** Binomial test between density dependent and independent models in terms of predictive ability.

Sites	Total number of time series	Density dependent	Density independent	p-value
ALF	968	465	503	p > 0.05
Bakker	78	25	53	p < 0.05
BBSALL	5591	1972	3619	p < 0.001
BBS10Stop	7450	2889	4561	p < 0.001
BSS	40	19	21	p > 0.05
DBBS	726	212	514	p < 0.001
Hay	37	14	23	p > 0.05
Portal	11	6	5	p > 0.05
PUP	354	120	234	p < 0.001
PUS	293	112	181	p < 0.001
PUY	121	45	76	p < 0.05
RM	338	154	184	p > 0.05
SCZ	49	22	27	p > 0.05
SNF	23	10	13	p > 0.05

The Chi-square test indicated that there was a significant difference in 12 of the 14 datasets among the four models in their predictive ability (Table 2, Supplementary Table 1). In none of the twelve cases does a density-dependent model make superior predictions statistically significantly more frequently than both density-independent models.

**Table 2:** Chi square significant test and pairwise comparison of the predictive ability of models in 14 datasets.

Datasets	Gompertz	Logistic	Mean	Trend
ALF	259^a^	206^b^	**365**^**c**^	138^d^
Bakker	17^a^	8^a^	**41**^**b**^	12^c^
BBSALL	1016^a^	956^a^	**2968**^**b**^	651^c^
BBS10STOP	1483^a^	1406^a^	**3895**^**b**^	666^c^
Buell-small (BSS)	**15**^**a**^	4^b^	**16**^**a**^	5^c^
DBBS	115^a^	97^a^	**391**^**b**^	123^c^
Hay	10^a^	4^d^	**21**^**b**^	2^dc^
Portal	3^a^	3^a^	4^a^	1^a^
Pumice plot	73^a^	47^b^	**221**^**c**^	13^d^
Pumice square	58^a^	54^a^	**165**^**b**^	16^c^
Pumice yard	24^a^	21^a^	**67**^**b**^	9^c^
Rothamsted	**88**^**abc**^	66^b^	**107**^**c**^	**77**^**ac**^
South central Ontario Zooplankton (SCZ)	**11**^**abc**^	**11**^**abc**^	**23**^**b**^	4^c^
San Nicolas fish (SNF)	4^a^	6^a^	10^a^	3^a^

NB: The values in each cell are the number of time series for that dataset that a particular model was identified as the best predictive model. For example: in ALF, the Gompertz model made the best predictions in 259 out of 968 time series. Values in bold are the most frequent or not statistically significantly different than the most frequent. Models that share the same letters are not statistically significantly different.

### Generalized linear mixed models

#### Absolute prediction error

For 7 out of 14 datasets, the ‘model’ term was not statistically significant. For the 7 datasets where the ‘Model’ term was statistically significant, neither of the density dependent models was superior to both of density independent models for any dataset (Table 3). For the seven datasets with a significant effect of model the Trend model resulted in significantly larger prediction errors than at least one of the other three models.

**Table 3:** Summary of statistical significance of the predictive ability of models for 14 datasets using generalized mixed modeling.

Sites	Models significance	Pairwise comparison
ALF	p < 0.001***	Gompertz^a^
		Logistic^a^
		Mean^a^
		Trend^b^
Bakker	p > 0.05	NA
BBSALL	p < 0.001***	Gompertz^a^
		Logistic^a^
		Mean^a^
		Trend^b^
BBS10Stop	p < 0.001***	Gompertz^a^
		Logistic^a^
		Mean^a^
		Trend^b^
DBBS	p < 0.001***	Gompertz^ab^
		Logistic^b^
		Mean^a^
		Trend^ab^
Hay	p < 0.001***	Gompertz^ab^
		Logistic^a^
		Mean^a^
		Trend^b^
BSS	p > 0.05	NA
Portal	p > 0.05	NA
PUP	p < 0.05**	NA^**^
PUS	p > 0.05	NA
PUY	p > 0.05	NA
RM	p > 0.05	NA
SCZ	p < 0.001***	Gompertz^a^
		Logistic^ab^
		Mean^ab^
		Trend^c^
SNF	p > 0.05	NA

NB: Models that share the same letters are not statistically significant.

NA^**^ in PUP despite the main effect being significant there were no pairwise comparisons that were statistically significant at the 0.05 threshold.

*indicates the significance difference in the predictive ability of the four models that do not share the same letter when compared to one another.

### Error bars illustrating variability around mean prediction error

The error bars represent the variability or dispersion around mean prediction error of the four models at the training set where each model has better performance. The trend model displays considerably higher variation around the mean prediction error in five datasets, while the other three models show relatively lower variation (Supplementary Fig. 1).

## Discussion

### Weak evidence for density dependent regulation

On average, the two simple density dependent models used here never made statistically significantly superior predictions than both density independent models. However, for four of 14 datasets, on average, a density dependent model made better predictions than either of the density independent models—just not statistically significantly so. It is worth noting that each of the 14 datasets is comprised of multiple time series so density-dependent models did have superior predictive performance for individual time series in a dataset. However, density-independent models had superior predictive performance more frequently than density-dependent models in 13 of 14 datasets and there was not a statistically significant difference for the one dataset where density-dependent models had superior performance more frequently. This suggests that density-dependent population regulation is relatively rare and/or weak.

### Empirical evidence for density dependent population regulation

The ecological literature has considerable evidence suggesting that both density dependent and density independent factors are important drivers of population fluctuations^31–36^. For example, Hanski^12^ concluded that 73% of moth species and 92% of aphid species showed evidence of density dependent regulation. Most studies examining multiple time series showed that 30–90% of time series showed evidence of density-dependent population regulation. By contrast, Gaston and Lawton^37^ did not find evidence of density dependent regulation for any of the 27 complete time series they analyzed. However, most studies used “fit to data” rather than predictive ability to detect or estimate the relative importance of density dependent factors.

Holyoak and Lawton^38^ detected density dependence in 10 (58.8%) of 17 taxa for series of 12 years and in 5 (33.3%) of 15 taxa with time series of 8 years in length. However, they used six different tests for density dependence and considered density dependence to have been detected if at least one of the six tests found evidence of density dependence. All of the tests used either statistical significance at the 0.05 threshold or permutation tests of the fit to the data. Their research led to further discussion on the literature addressing the issue of ecological density dependence (EDD) versus statistical density dependence (SDD). That is, evidence that time series show a return tendency (SDD) versus evidence that ${N}_{t}$ has a causal effect on ${N}_{t+1}$ (EDD). Wolda, Dennis and Taper^39^ provided a compelling case that statistical evidence of density dependence provides only weak evidence of ecological density dependence.

Woiwod and Hanski^40^ used a much larger dataset of exclusively moths and aphids but only three rather than six tests of density dependence. Again, each of the tests used a statistical significance threshold of 0.05 to conclude whether there was evidence of density dependence. There was large variation among tests, sites and species in statistically significant evidence for density dependent regulation, but about 30–50% of time series showed statistically significant evidence for density dependence across all three tests.

Moreover, Brook and Bradshaw^41^ took a similar approach as Holyoak and Lawton^38^ but with a much larger dataset. They used several tests for density dependence, (1) multi-model inference comparing two density independent models (i.e. random walk and exponential) and three density dependent models (the logistic, Gompertz and theta-logistic) and using AIC weights to assign the relative strength of evidence (Burnham and Anderson, 2004), (2) BIC^42^ and C-V^15^ model selection and (3) null hypothesis significance testing model selection^24,43,44^. They concluded that 74.7% of 1198 populations showed evidence of density dependent population regulation using multi-model inference. Cross-validation indicated that 93.9 % of population showed evidence of density dependent regulation. Null hypothesis Statistical testing (NHST) approaches were more conservative suggesting that 30–50% of populations were density dependent. The evidence for the Gompertz model was twice as strong as for the logistic model. They also provided evidence that density dependent population regulation was more easily detected in longer time series.

### Comparison of our study to previous research

Our study differed from previous studies in four important ways. First, we used prediction error rather than model fit to infer evidence of density dependent population regulation. Second, we used datasets containing multiple time series so that we had ‘within dataset’ replication. Third, we used a ‘moving window’ approach to building training sets so that we had ‘within time-series’ replication. Fourth, we had a much larger number of time series than any previous study.

In general, our results were consistent with studies that showed density dependent population regulation was weak or uncommon in most ecological communities though evidence for density dependent population regulation varied across types of ecological communities (i.e., “across dataset” differences) and across populations within particular ecological communities (i.e., “within dataset” differences). Consistent with previous studies we also found that when density dependent regulation was detected the Gompertz model was superior to the logistic model.

However, our results for predictive ability could be interpreted in two ways. First, the fact that for none of the datasets was either density dependent model statistically significantly superior to both of the density independent models for absolute prediction error, implies that there is no evidence that density dependent regulation is stronger or more common than we would expect by chance. However, it might be that some populations are strongly density dependent, and others are not density dependent at all, but that we don’t see an ‘average’ effect because one offsets the other.

It is also important to note that we used two simple density dependent models, the logistic model and the Gompertz model, and it is possible that models incorporating density-dependence in different ways would have made better predictions than the logistic and Gompertz models and better predictions than the density-independent models. In addition, these two models only incorporate density-dependent effects on overall population size and density-dependence can affect many other characteristics of a population including abundance at different life stages, body size and sex ratio^45–47^.

## Summary

Identifying the key drivers of population fluctuations, including estimating the relative importance of density-dependent factors, is important in managing natural populations and one of the fundamental questions in ecology. Here, we’ve used predictive ability to evaluate two simple density-dependent models and two density-independent models and found only weak evidence of density dependent regulation of population size in natural populations. However, a comprehensive assessment of the relative importance of density-dependent population regulation will require extending the analysis to estimating the predictive ability of a larger suite of density dependent model, state-space models that explicitly incorporate both process and observation error and models that include other potentially important drivers such as climate and biotic interactions. In addition, we will need to assess the density-dependence of endpoints other than total population size.

## Methods

### Dataset selection

We selected population time series data from 14 different databases (Supplementary Table 2). These datasets were from multi-sites, multi-year, and multi-species abundance/density databases. We chose only databases with at least 20 consecutive years of surveyed data. We also used only population time series from each database with abundance/density greater than zero for each of the years in the time series.

### Data description

The datasets used in this analysis were from North America (10 out of 14), Canada (1 dataset), The Netherlands (2 datasets), and United Kingdom (1 dataset). Three aquatic ecosystems and eleven terrestrial ecosystems were represented in the datasets. Types of organisms under study were birds, plants, zooplankton, rodents, fish and moths. The minimum and maximum number of years in a time series is 20 and 30 years respectively. Thus, the maximum number of years in a training set ranged from 11 to 21 years (see section detailed descriptions of training sets and testing sets below). The number of time series in the datasets ranged from 11 to 7450. The mean number of different species in a dataset was around 60 and range of the number of different species was 3–254. The mean number of different sites in a dataset was 53 and the range in the number of different sites was 4–283. Sampling methods included trawl surveys, visual searches, live trapping and quadrat mapping. The units of abundance/density estimates include number of individuals per 1 ${m}^{2}$ plot, number of individuals per ${m}^{2}$ and percent cover).

### Using data to fit models

We fitted four (4) different models to each time series in each of the 14 datasets. The models were fit to a training set to estimate parameters then the fitted models were used to predict values in the test set.

### The models

Dennis and Taper (1994)^24^ in a classic series of density dependent papers used the logistic equation to identify four plausible models which are still considered among the best density dependent models. These models have never been assessed in the context of their predictive ability. We used the same four models as follows.

#### Mean model

1 $$log\left(\frac{{n}_{t+1}}{{n}_{t}}\right)=b+m\left({n}_{t}\right) ,\, b, \,m=0$$

#### Trend model

2 $$log\left(\frac{{n}_{t+1}}{{n}_{t}}\right)=b+m\left({n}_{t}\right) ,\, b\ne 0,\, m=0$$

#### Logistic model

3 $$log\left(\frac{{n}_{t+1}}{{n}_{t}}\right)=b+m\left({n}_{t}\right) ,\, b\ne 0,\, m\ne 0$$

#### Gompertz model

4 $$log\left(\frac{{n}_{t+1}}{{n}_{t}}\right)=b+m\left({\text{ln}}({n}_{t})\right) ,\, b\ne 0,\, m\ne 0$$

The key difference between density dependent (Eqs. (3) and (4)) and density independent models (Eqs. (1) and (2)) is that the slope in density dependent models is different from zero. Each of the four models was fit to each training set for each time series from each dataset.

### Detailed descriptions of training sets and testing sets

To assess the predictive ability of the population models, the time series data were partitioned into training and testing sets.

*Step 1:* We selected the last nine years as the test set and the first N-9 years as the training set. Because the number of years in time series ranged from 20 to 30 years among the datasets, this means that not all training sets across datasets had the same number of years although within each dataset the number of years in each training set was constant.

**Figure 3:**
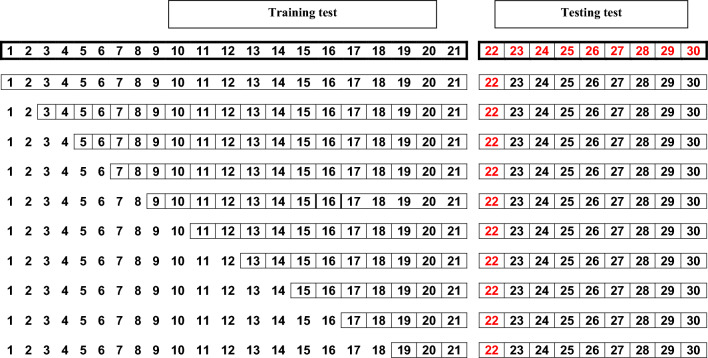
The following grid shows the sliding window to get training sets of varying lengths.

*Step 2:* Because we wanted to assess the optimum number of years to include in a training set, we subset the ‘complete’ training set into a series of training sets containing 1, 3, 5…. N-9 years. For example, for a dataset containing time series that were 30 years long, the ‘complete’ training set would include the first 21 years. The ‘complete’ training set would then be subset into training sets of 1, 3, 5, 7, 9, 11, 13, 15, 17, 19, and 21 years (Fig. 3). Subsets would be created by excluding early years. Thus, if a training subset was to only include three years of data, they would be the years 19–21. Data were fit to each of the four models using each of the subsets with the exception of models that could not be fit using a single year (i.e., both density dependent models and the trend model) or year interval (i.e., both density dependent models). In the example for a dataset with 30 years of data, this implies that there would be 10 or 11 sub models (i.e., one for each of the training subsets) for each of the four models.

*Step 3:* All models were used to predict the year immediately after the last year in the training subset. So, for all subsets of a ‘complete’ training set that included year 21, year 22 would be the predicted population size.

**Figure 4:**
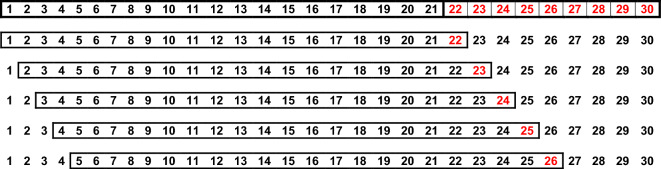
The following grid shows the moving window to get 5 comparisons between observed and predicted values for each training length.

*Step 4*: “*Rolling window*”: To maximize the number of predictions available for assessing predictive ability, the training and testing sets were chosen from the time series in a ‘rolling window’ method (Fig. 4). This allowed us to compare 5 times as many predicted values to the observed values but still ensures that the model predictions were tested on data that were not used to train the models. The ‘complete’ training set for each database was shifted one year forward in each database and Steps 2–4 was repeated. For example, for a database with a 30-year time series that has used years 1–21 for the initial ‘complete’ training set, the training set would be shifted on year forward to include year’s 2–22. This implies that the test set will shrink to eight years in length because there would only be eight years remaining to include in the test set. Now, instead of using models trained on years 1–21 to predict years 22, we used models trained on years 2–22 to predict year 23.

Note that prediction errors within the rolling window may not be independent because the training sets used to estimate model parameters will have many data points in common and the ‘test’ data will all be within a 5-year window. However, we use means of the five data points in the ‘rolling window’ in all of our analyses and aren’t making any statistical inferences based on variability among those 5 data points.

*Step 5:* We repeated the shift of the ‘complete’ training set forward one year three more times until the available tests set only includes the last five years in the time series. This allowed us to make five replicate predictions for each model/training set combination for each time series in each dataset. For example, for a dataset containing 30 years of data, there were five replicate predictions for the Gompertz model using the 21-year training set to fit a model and predict one year out because we used years 1–21 to predict year 22, years 2–22 to predict year 23, years 3–23 to predict year 24, years 4–24 to predict year 25, and years 5–25 to predict year 26.

### Mean prediction error

#### Absolute

We calculated absolute prediction error for a single prediction as |Observed-Predicted|. Mean prediction error was calculated separately for each model/training set size combination. For example, a dataset containing time series of 30-year duration, would calculate a different mean for the Gompertz 21-year training set, the Gompertz 19-year training, the Gompertz 17-year training set and so on. The mean is calculated across all times series and all 5 replicate predictions for each time series.

5 $$\mathrm{Mean \,Absolute \,Prediction \,Error }=\sum_{1}^{N=\# \,of\, time\, series}\sum_{1}^{n=5}|Absolute\, Prediction \,Error|/N*n,$$

Where N = number of time series in a dataset (e.g., 968 for Aleutian Island Fish dataset), n = number of replicates for each model (training set combination = 5), and the prediction error is the difference between the observed and predicted values for a specified replicate of a particular model times series—training set combination. For example, the Aleutian Island Fish dataset has 968 time series and there would be 5 replicate predictions for each model—time series combination for a total of 968 × 5 = 4840 predictions for each model—training set combination. That is, the Gompertz model fits with 19 years in the training set would have 4840 predictions and therefore 4840 prediction errors.

### Statistical analyses

#### Model effects

To test for differences in predictive ability among models (i.e., Gompertz, Logistic, Mean and Trend), we used a linear mixed model with a fixed effect for the model term and random effects for the site and species. For statistical comparisons we always compared predictive ability among the four models using the training set size that had the lowest mean prediction error a particular model. For example, the statistical comparison of the four models for the Aleutian Islands Fish dataset used 17 years of training data for each of the ‘Trend’, ‘Logistic’, and ‘Gompertz’ models and 5 years for the ‘Mean’ model. This was done separately for each of the 14 datasets.

$${\text{Prediction error }}\sim {\text{ Models }} + \, \left( {{1}|{\text{Sites}}} \right) \, + \, \left( {{1}|{\text{Species}}} \right).$$

### Binomial and Chi-square test for density dependent and independent models

The statistical test in model effects tests for differences in mean prediction error among models but we also tested for statistically significant differences in the frequency that each model was ‘best’ (i.e., made the smallest prediction error). We used a binomial test to test whether the frequency that density dependent models were best was significantly different than the frequency that density independent models were best. Moreover, we used a Chi-square test to test for a difference in frequency among the four models (Gompertz, Logistic, Mean and Trend).

### Supplementary Information

Supplementary Information 1.

Supplementary Information 2.

## Data Availability

All data analysed during this study are included in the supplementary information files.
