# Supplementary material for: Weak evidence of density dependent population regulation when using the ability of two simple density dependent models to predict population size
Source: Sci Rep. 2024 Feb 29;14:5051. doi: 10.1038/s41598-024-55533-4 (PMC10904816; doi:10.1038/s41598-024-55533-4)
Supplement: Supplementary file 2 — Supplementary Information 2. [file 41598_2024_55533_MOESM2_ESM.docx]

**Supplementary information**

**Supplementary figure 1: Error bars illustrating variability of mean prediction error at the best performance of the four models for 14 datasets**


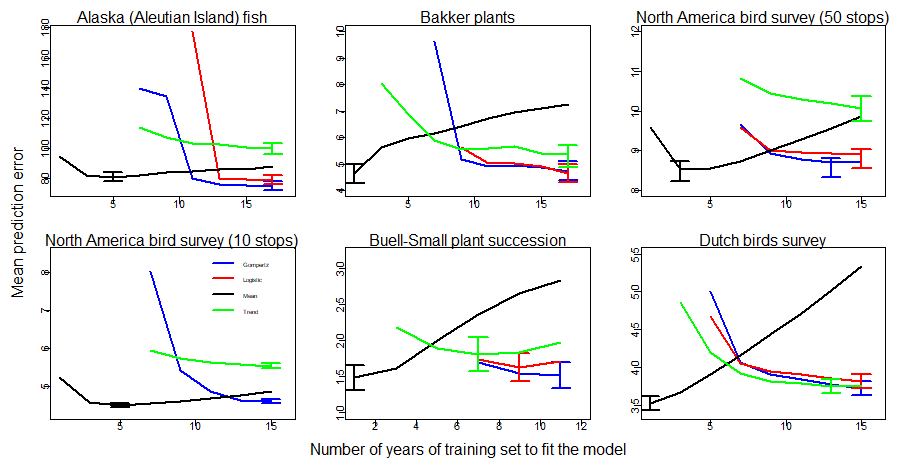


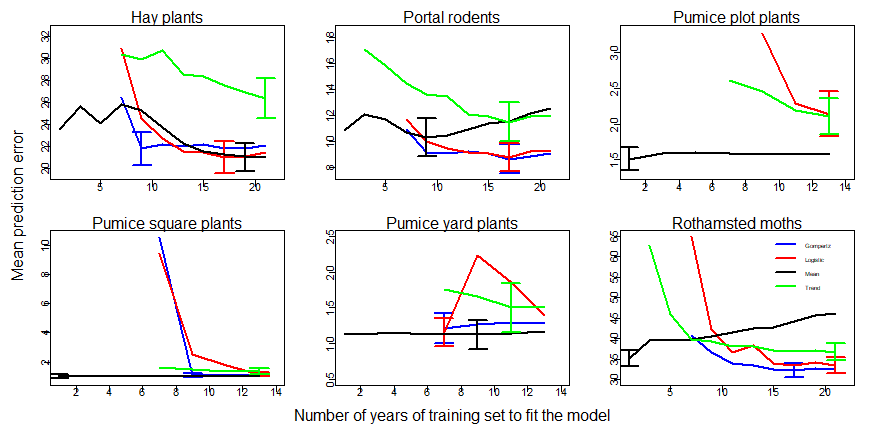


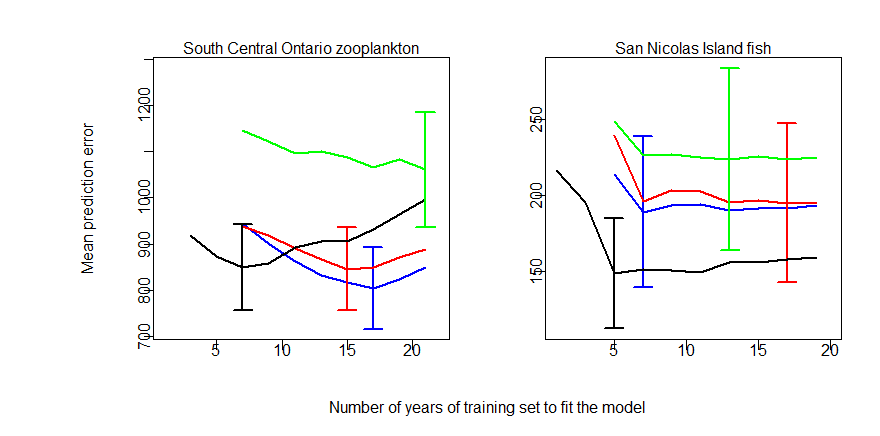


- The error bars in the graph illustrate the variability around the mean prediction error, indicating the performance of each model in the corresponding datasets. The standard error of the mean (SE) is used to quantify this variability.

**Supplementary figure 2: Mean absolute prediction error using ‘best’ training set size with error bars for each of the 14 datasets
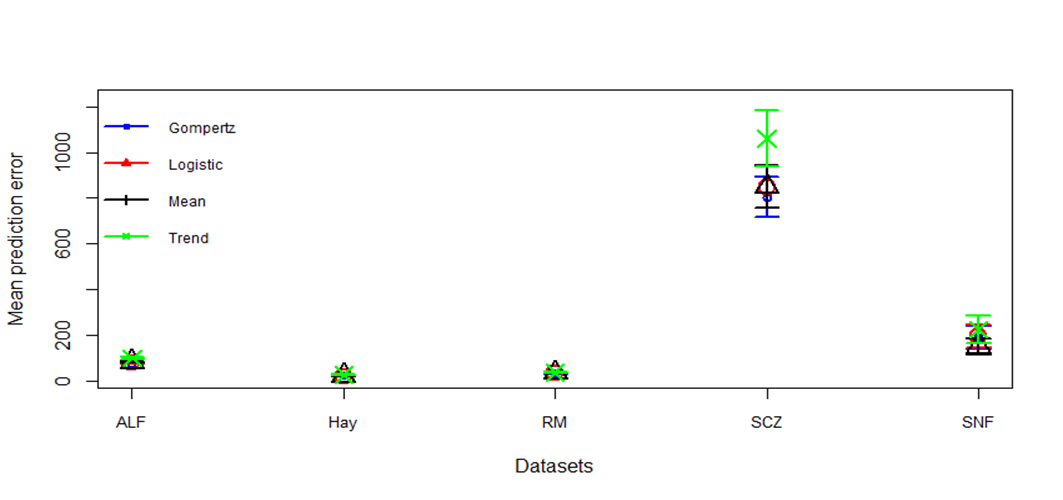

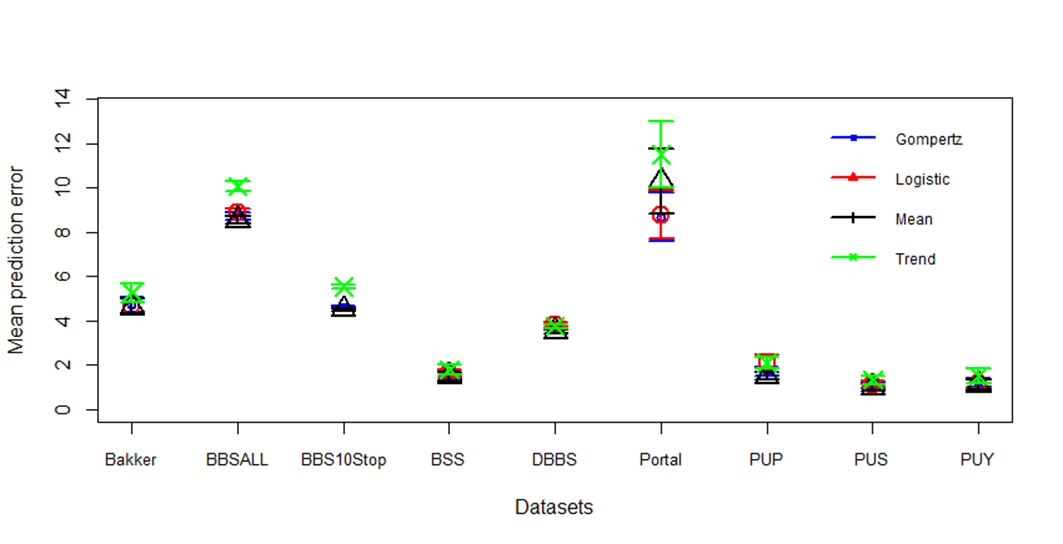
**

**Supplementary table 1:** Chi square significant test and pairwise comparison of the predictive ability of models in 14 datasets

| **Sites** | **Gompertz** | **Logistic** | **Mean** | **Trend** | **Total number of time series** | **p-value** |
| --- | --- | --- | --- | --- | --- | --- |
| ALF | 259 | 206 | 365 | 136 | 968 | p < 0.001 |
| Bakker | 17 | 8 | 41 | 12 | 78 | p < 0.001 |
| BBSALL | 1016 | 956 | 2968 | 651 | 5591 | p < 0.001 |
| BBS10Stop | 1483 | 1406 | 3895 | 666 | 7450 | p < 0.001 |
| BSS | 15 | 4 | 16 | 5 | 40 | p < 0.05 |
| DBBS | 115 | 97 | 391 | 123 | 726 | p < 0.001 |
| Hay | 10 | 4 | 21 | 2 | 37 | p < 0.001 |
| Portal | 3 | 3 | 4 | 1 | 11 | p > 0.05 |
| PUP | 73 | 47 | 221 | 13 | 354 | p < 0.001 |
| PUS | 58 | 54 | 165 | 16 | 293 | p < 0.001 |
| PUY | 24 | 21 | 67 | 9 | 121 | p < 0.001 |
| RM | 88 | 66 | 107 | 77 | 338 | p < 0.05 |
| SCZ | 11 | 11 | 23 | 4 | 49 | p < 0.05 |
| SNF | 4 | 6 | 10 | 3 | 23 | p > 0.05 |

- Table showing the number of time series where each model outperformed others across the total time series for each 14 datasets and its significance assessment.

**Supplementary table 2: Description of 14 datasets used in the research project.**

| **Datasets** | **Abbreviation** | **Location** | **Group** | **Number of**  **Species** | **Time series** | **Number of Training Set Years** |
| --- | --- | --- | --- | --- | --- | --- |
| Aleutian Islands Ground Fish Survey | ALF | Alaska, US | Fish | 16 | 968 | 17 |
| Bakker Plant | Bakker | Netherlands | Plants | 22 | 78 | 17 |
| North America Breeding Bird Survey (50 stops) | BBSALL | North America | Birds | 254 | 5591 | 15 |
| North America Breeding Bird Survey (10 stops) | BBS10STOP | North America | Birds | 195 | 7450 | 15 |
| Buell-Small Succession Study | BSS | New Jersey, USA | Plants | 18 | 21 | 11 |
| Dutch Breeding Bird Survey | DBBS | Netherlands | Birds | 103 | 726 | 15 |
| Hay Plants | Hay | Kansas, USA | Plants | 8 | 37 | 21 |
| Portal Rodents | PR  Portal/PR | Arizona, USA | Rodents | 3 | 11 | 21 |
| Pumice Plot | PUP | Mt. St. Helens, USA | Plants | 16 | 344 | 13 |
| Pumice Square | PUS | Mt. St. Helens, USA | Plants | 18 | 293 | 13 |
| Pumice Yard | PUY | Mt. St. Helens, USA | Plants | 20 | 121 | 13 |
| Rothamsted Moths | RM | UK | Moths | 144 | 338 | 21 |
| South Central Ontario Lakes Zooplankton | SCZ | South Central Lakes, Canada | Zooplankton | 14 | 49 | 21 |
| San Nicolas Island Fish | SNF | California, US | Fish | 10 | 23 | 19 |
